# Navigating glioblastoma complexity: the interplay of neurotransmitters and chromatin

**DOI:** 10.1007/s11033-024-09853-3

**Published:** 2024-08-17

**Authors:** Jessica Romero-Reyes, Edgar Ricardo Vázquez-Martínez, Carlos-Camilo Silva, Anayansi Molina-Hernández, Néstor Fabián Díaz, Ignacio Camacho-Arroyo

**Affiliations:** 1 Unidad de Investigación en Reproducción Humana, Instituto Nacional de Perinatología-Facultad de Química, Universidad Nacional Autónoma de México, Mexico City, México; 2 Chronobiology of Reproduction Research Lab. Biology of Reproduction Research Unit, Carrera de Biología, Facultad de Estudios Superiores Zaragoza, Universidad Nacional Autónoma de México, Mexico City, México; 3 Departamento de Fisiología y Desarrollo Celular, Instituto Nacional de Perinatología, Mexico City, México

**Keywords:** Glioblastoma, Glioma stem cells, Neurotransmitters, Post-translational modifications, Serotonylation

## Abstract

Glioblastoma is the most aggressive brain cancer with an unfavorable prognosis for patient survival. Glioma stem cells, a subpopulation of cancer cells, drive tumor initiation, self-renewal, and resistance to therapy and, together with the microenvironment, play a crucial role in glioblastoma maintenance and progression. Neurotransmitters such as noradrenaline, dopamine, and serotonin have contrasting effects on glioblastoma development, stimulating or inhibiting its progression depending on the cellular context and through their action on glioma stem cells, perhaps changing the epigenetic landscape. Recent studies have revealed that serotonin and dopamine induce chromatin modifications related to transcriptional plasticity in the mammalian brain and possibly in glioblastoma; however, this topic still needs to be explored because of its potential implications for glioblastoma treatment. Also, it is essential to consider that neurotransmitters’ effects depend on the tumor’s microenvironment since it can significantly influence the response and behavior of cancer cells. This review examines the possible role of neurotransmitters as regulators of glioblastoma development, focusing on their impact on the chromatin of glioma stem cells.

## Introduction

Glioblastoma (GB) is the most aggressive brain cancer, mainly derived from astrocytes or glioma stem cells [(GSCs), a type of cancer stem cell (CSC)] [1, 2], which are responsible for resistance to GB treatment [3–5]. GB patients have a poor survival prognosis, ranging from 12 to 15 months, despite the treatment, which includes surgical removal of the tumor followed by radiotherapy and chemotherapy with temozolomide [(TMZ), an alkylating agent] [4, 5]. This pathology is 1.6 times more frequent in men than in women [6–9].

GB tumors have been classified into four categories with particular gene signatures: (1) classical, overexpressing genes such as *EGFR, FGFR3, PDGFA, AKT2*, and *NES*; (2) neural, characterized by *FBXO3, GABRB2, SNCG,* and *MBP* overexpression; (3) proneural, with *DLL3, NKX2-2, SOX2, ERBB3*, and *OLIG2* overexpression, and (4) mesenchymal, characterized by *CASP1/4/5/8, ILR4, CHI3L, TRADD, TLR2/4*, and *RELB* overexpression [10–12].

In malignant tumors of the central nervous system (CNS), neurotransmitters such as noradrenaline (NA), dopamine (DA), and serotonin (5-HT) have been identified [10, 13]. These neurotransmitters are possibly supplied to the tumor through innervation, given that several types of cells that constitute the GB tumors, such as glial cells (astrocytes, oligodendrocytes, and microglia), express synaptogenic proteins [14–16]. Supporting this hypothesis, the distribution of these neurotransmitters is higher in the peripheral regions of malignant brain tumors than in the central area [13]. Also, local synthesis in GB may exist due to the enzymes that participate in the catabolism of tyrosine and tryptophan have been detected [10].

In the GB context, neurotransmitters influence cellular metabolism through several pathways. The treatment of GB cells with NA inhibits glucose uptake and, at the same time, stimulates its release; these events are accompanied by the increase in 3′:5′-cyclic AMP and glycogen breakdown [17]. Meanwhile, DA increases the glucose reuptake in GB cell lines and induces apoptosis by stimulating the DRD2 receptor [18, 19]. Interestingly, the exposure of GB cells to fluoxetine (FLX), a selective 5-HT reuptake inhibitor (SSRI), remodels the sphingolipid distribution in the plasma membrane of GB cells, increasing the annexin V appearance, which is an indicator of intermediate stages of apoptosis [20]. Thus, these neurotransmitters play a pivotal role in the GB niche, regulating cellular proliferation, migration, apoptosis, metabolism, survival, differentiation, and angiogenesis [21].

The chromatin status plays a crucial role in the progression of GB [22]. The nucleosome is the basic structural unit of DNA packaging in eukaryotes, consisting of a core of histone proteins (H2A, H2B, H3, and H4), forming an octamer around 147 DNA base pairs. The amino acid residues at the N-terminus of the histones can be covalently modified by processes such as acylations, ubiquitin-like modifications, methylation, biotinylation, ADP ribosylation, and phosphorylation [23, 24]. Likewise, histone variants such as H2AX, H2AZ, and H3.3 regulate DNA repair, sex chromosome remodeling, nucleosome stability, and the activation of regulatory elements and gene expression [25].

In pediatric and young adult GB, mutations in H3F3A (a variant of H3 histone) are recurrent, with amino acid substitutions in histone tails K27M or G34R/G34V [26]. Moreover, several patient tumors harbor mutations of H3-K27M in oligodendroglial precursor-like cells, which are believed to be the origin of these tumors [27, 28].

Recently, it has been described that 5-HT and DA are chromatin modifiers [29, 30]. These neurotransmitters can be covalently bound to glutamine-5 or 19 on histone 3 (H3Q5/19) through the action of transglutaminase 2 (TGM2) [29, 31]. Post-translational modifications (PTMs) induce changes in gene expression, and in the case of 5-HT, they are permissive to gene expression, while in the case of DA, they alter gene transcription [29, 30]. Despite the widespread distribution of these neurotransmitters in the brain during normal and pathological conditions, their potential role in GB, especially in GSCs, remains poorly described. This review examines the role of NA, DA, and 5-HT as regulators of GB progression. Additionally, we explore their potential as chromatin modifiers in GSCs, a role minimally described in the literature.

## Glioma stem cells

GBs have multiple GSC populations distributed in several tumor niches [32], including the perivascular niche (PVN), characterized by abnormal angiogenesis. The hypoxic niche, in which necrotic cells prevail, often results from vascular regression. This environment experiences an upregulation of hypoxia-inducible factor (HIF) [33], which is favorable for the self-renewal of GSCs [34]. Another niche is the invasive GB, which extends into surrounding tissues and is typically located at the border of normal brain parenchyma. It exhibits a more functional vasculature than PVN and possesses cellular heterogeneity [32, 33].

Furthermore, GSCs are characterized by the expression of several markers, including surface antigens, intermediate filaments, and transcription factors such as Prominin-1 (PROM1) or CD133, CD15, Nestin, and SOX2 [4, 35, 36]. Likewise, GSCs exhibit the capacity for self-renewal and differentiation into neurons, astrocytes, and oligodendrocytes [37, 38]. In addition, GSCs can express neural progenitor (*SOX4, OLIG2*, and *ASCL1*), astrocytic (*GFAP, APOE, AQP4, CD44, CD9*, and *VIM*), or neuronal marker genes (*CD24, SOX11*, and *DCX*) [39].

GSCs exhibit a proliferating or slow cycling/quiescent behavior in GB [40, 41]. In GB tumor slices, CD133 was expressed in 45% of the cells that were positive for Ki67, while Nestin was expressed in 20% [41]. Interestingly, in a forebrain organoid model generated with human induced pluripotent stem cells (hiPSCs), with tumors formed by injecting GB7 or COMI GB cell lines, Ki67 (a proliferation marker) was absent. At the same time, there was an increase in SOX2 expression, a marker for GSCs [42]. Regarding GSCs’ quiescent behavior, an F3 cell-surface receptor mRNA was recently identified as a conserved signature whose expression induction increases cellular self-renewal [43]. Furthermore, when dissociated tumoral organoid cells were injected into CD1-Nude mice brains, the resulting tumors exhibited a similar expression pattern of Ki67 and SOX2 as the inoculated organoids to the GB cell lines [42]. These findings support the notion that GSCs are a heterogeneous population with proliferating or quiescent behavior [44]; also, the GSCs share epigenetic mechanisms with neural stem cell (NSC) [45] characteristics that may play a role in maintaining their population despite GB treatments.

In addition, GSCs display changes in chromatin reorganization and remodeling, which are critical for maintenance and tumorigenicity. PTMs often drive these changes. In cultures of human GSCs, three clusters have been characterized by the presence of the trimethylation of Histone H3 Lysine 4 (H3K4me3) and Histone H3 Lysine 27 acetylation (H3K27ac) in active promoter regions of genes as *P2RY1, MAPT, OLIG2*, and *ASCL1* (cluster 1), H3K27ac in promoter regions of *CD44, ACVR1, RUNX1* and *TGFBR2* (cluster 2), and H3K4me1 in the enhancer region of *SOX5* (cluster 3). It is known that the expression of these genes confers GSCs’ highly proliferative and migratory characteristics (Table 1) [46–48].

**Table 1:** Histone post-translational modifications (PTM) associated with gene expression enrichment in glioma stem cells related to different brain lineages

Histone/PTM	Gene Signature	Lineage	References
H3K4me3, H3K27ac	*SOX4, OLIG2, ASCL1, MAPT, P2RY1*	Neural progenitors	[28, 33, 34]
H3K27ac	*GFAP, APOE, AQP4, CD44, CD9, VIM, ACVR1, RUNX1, TGFBR2*	Astrocytic/Oligodrendrocytic	[28, 33, 36]
H3K4me1	*CD24, SOX11, DCX, SOX5*	Neuronal	[28, 33]

Moreover, histone variants are incorporated into specific genomic regions throughout the cell cycle [49]. Regarding histone variants, it has been shown that H2A.Z is overexpressed in GSCs and is crucial for their maintenance [50].

## The role of chromatin modification H2A.Z in neoplasias development: the prospect in glioblastoma

H2A.Z is an evolutionarily conserved H2A histone variant that shares 60% of its amino acid sequence with canonical H2A. It is related to nucleosome stability, either increasing or decreasing its mobility, which is influenced by histone variants and PTMs. Typically, this histone variant is found in promoters and enhancers. Also, the variant H2A.Z colocalizes with PTMs such as H4ac, H3K4me3, H3K4me1, and H3K27me3 [51].

H2A.Z is involved in the epithelial-mesenchymal-transition (EMT) [51–53] and has been related to the mechanisms involved in cancer development, such as mitosis and cellular metabolism [54, 55]. In the epithelioid pancreatic carcinoma PANC-1 cell line, the histone H2A.Z is enriched in the active gene promoters related to H3K4me3, H3K27ac marks and in gene enhancers associated with H3K27ac and H3K4me1 marks, as observed by chromatin immunoprecipitation with sequencing (ChIP-seq) analysis [56]. Pathway analysis of H2A.Z enrichment in enhancers identified genes related to focal adhesion, Wnt signaling, and HIF-1 transcriptional activity in hypoxia (among others). It is important to emphasize that HIF expression is associated with the presence of stemness markers, potentially contributing to the maintenance of the CSCs population and, consequently, cancer.

In the human LD611 cell line, derived from bladder cancer, the presence of H2A.Z is higher than in human UROtsa cells, a non-malignant uroepithelium line. When the 3–4, 5-(dimethyl-thyazol-2-yl)-2,5-diphenyltetrazolium (MTT) assay was performed, the existence of this histone was related to cellular proliferation in the LD611 cell line. In general, H2A.Z is located adjacent to the transcription start sites (TSS) of genes involved in cell cycle regulation, cellular development, cell growth, proliferation, cell death, and survival [57].

Analysis of the TCGA and Repository for Molecular Brain Neoplasia Data (REMBRANDT) datasets reveals an association between high expression of H2A.Z and poor prognosis for GB patient survival. Furthermore, a positive correlation between this histone and CD133^+^ or Nestin^+^ cells in GB has been observed. Studies conducted in a 3D model enriched with GSCs demonstrate that silencing of H2A.Z results in the formation of smaller-sized spheres. Similarly, smaller tumors are formed when cells from these spheres are injected into the brains of immunosuppressed NOD-scid IL2Rgamma^null^ mice (NSG™), compared to those formed with cells expressing intact H2A.Z. The enrichment of H2A.Z in the epigenetic landscape of GSCs is associated with colocalization with H3K27ac, a marker related to chromatin transcriptional activity. This suggests that H2A.Z regulates the accessibility of transcriptional regulators to enhancer elements within GSC gene promoters [50].

**Fig. 1:**
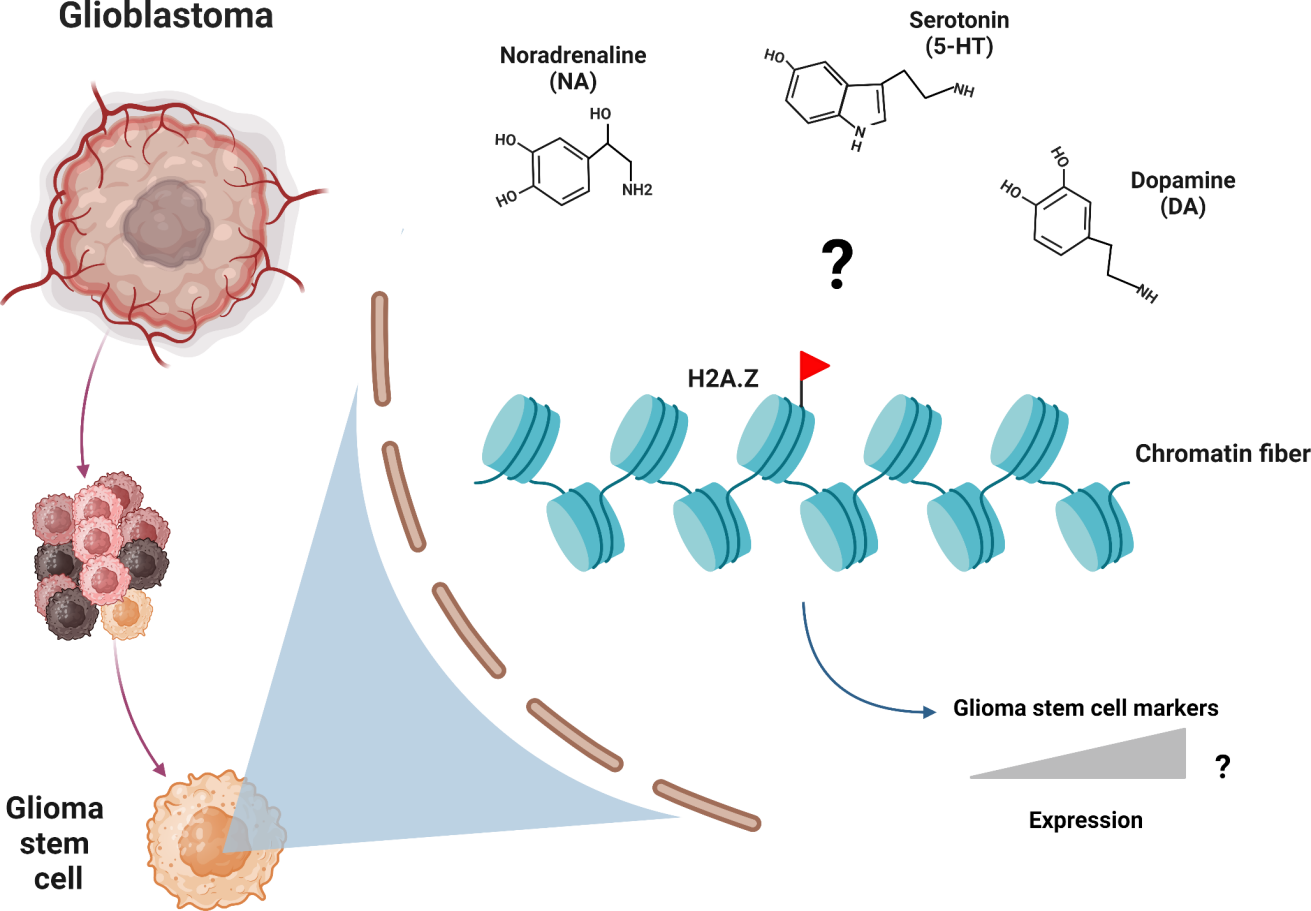
Potential chromatin regulation of glioma stem cells by neurotransmitters. Glioblastoma tumors are integrated by distinct cellular types, including glioma stem cells (GSCs). It has been hypothesized that H2A.Z is susceptible to modification by 5-HT and DA, and possibly NA, which could stimulate the expression of glioma stemness markers. The figure was created with BioRender.com

H2A.Z, as demonstrated by the DNA-barcoded nucleosome libraries technique, is susceptible to modification by tissue transglutaminase 2 (TGM2) [31], an enzyme responsible for depositing 5-HT and DA on histones [29–31]. This discovery prompts further investigation into whether this mechanism occurs in GSCs and its implications for their biology (Fig. 1).

This rationale encourages exploration into the role of neurotransmitters known for their chromatin remodeling function within the CNS. Such investigations may provide crucial insights into the potential regulatory mechanisms governing GSCs’ behavior, offering a foundation for targeted therapeutic interventions and advancing our comprehension of the intricate relationship between neural signaling and chromatin modulation in GB.

## Neurotransmitters microenvironment in glioblastomas and their potential role in chromatin remodeling

### Noradrenaline

NA enhances human GB-derived cell lines (U87 and U251) metabolism, migration, and invasion [58]. Moreover, the Human Tumor Metastasis RT2 Profiler PCR Arrays showed that exposure to NA diminishes the presence of matrix metallopeptidase-11 (MMP-11), an enzyme related to the breakdown of the extracellular matrix whose expression is higher in GB than in non-malignant-brain-tumors [59, 60].

**Fig. 2:**
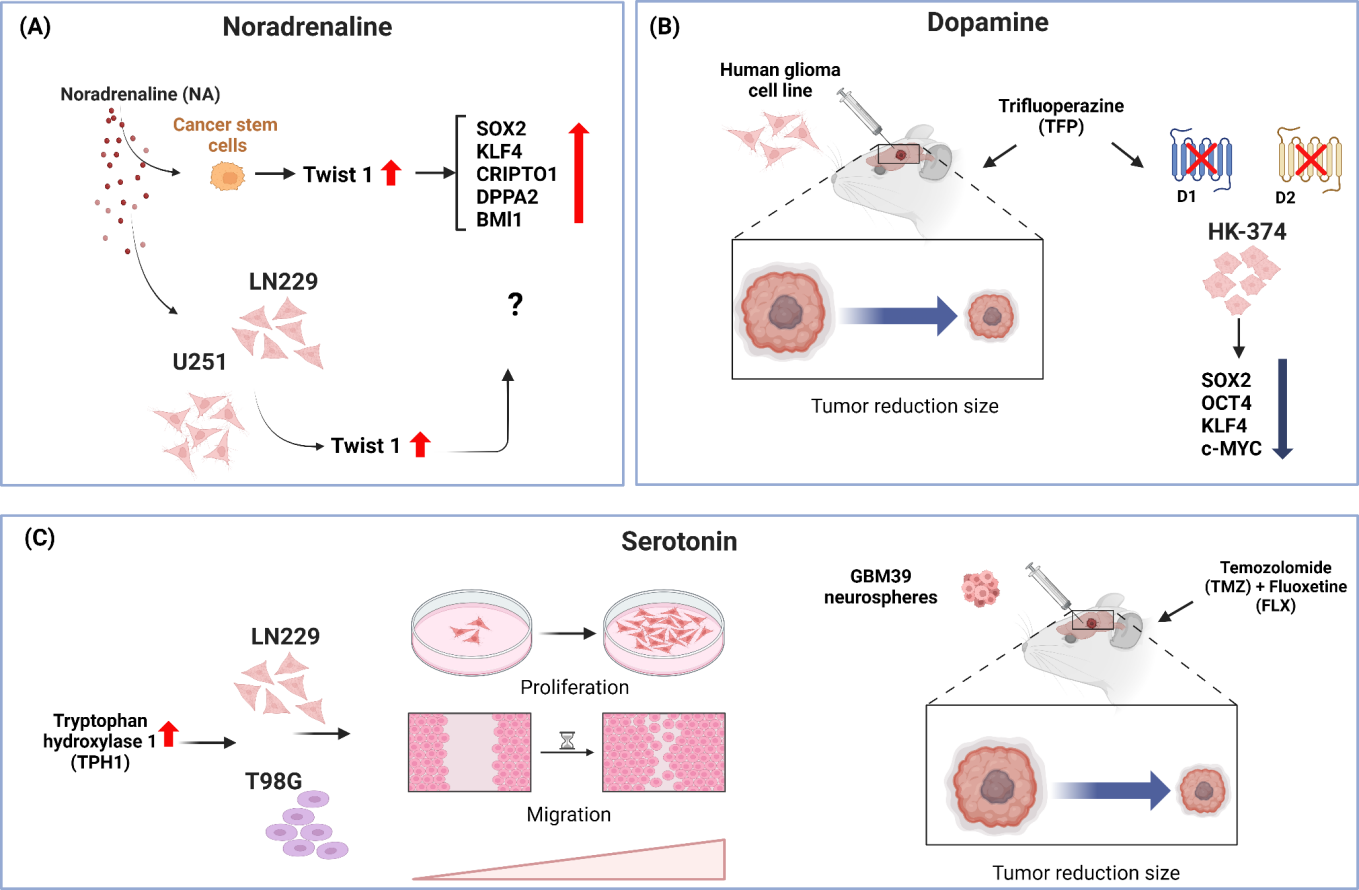
Role of noradrenaline, dopamine, and serotonin in Glioblastoma progression. (A) Exposure of CSCs to NA enhances *Twist 1* expression. This increment is related to the increase in *SOX2, KLF4, CRIPTO1, DPPA2*, and *BMI1* expression, considered “stemness markers.” Also, the culture of human-derived glioblastoma cell lines LN229 and U251 with NA increases *Twist 1* expression. (B) In glioblastoma orthotopic models, the use of TFP, an antagonist of dopamine receptors 1 (D1) and 2 (D2), is related to tumor size reduction and a decrease in *SOX2, KLF4*, and *c-MYC* expression, which are transcription factors characteristic of stemness. (C) The stimulus of TPH1 expression, the rate-limiting enzyme in serotonin synthesis, in the LN229 and T98G glioblastoma cell lines promotes cellular migration and proliferation. In contrast, exposure to TMZ, an alkylating drug commonly used in the treatment of glioblastoma, plus FLX, a selective inhibitor of serotonin reuptake, of tumors induced by neurospheres from the GBM39 glioblastoma cell line, resulted in the reduction of tumor size. The figure was created with BioRender.com

In 2022, Wang and colleagues showed that NA stimulates EMT in GB cells, a mechanism for migration and metastasis. They analyzed The Glioma French, the Cancer Genome Atlas (TCGA), and the Chinese Glioma Genome Atlas datasets, reporting that the expression of Twist 1 [a gene that stimulates the stemness markers such as *BMI1, CRIPTO1, DPPA2, KLF4*, and *SOX2* in CSC [61]] is related to a poor prognosis in GB patients. Interestingly, when the human-derived-GB cell lines (U251 and LN229) were cultured in the presence of NA, an increase in *Twist 1* expression was observed (Fig. 2). In contrast, the knock-down of *Twist 1* by shTwist 1 lentivirus diminishes the presence of N-cadherin, fibronectin 1, and vimentin, EMT markers [62].

Interestingly, in the context of the murine brain, it has been hypothesized that NA could be a chromatin modifier [63]. When hippocampal slices of male C57BL/6 strain mice brains were treated with NA, after dissecting the CA1 region and extracting the histones, it was observed that H3K14ac was associated with EMT [63]. Possibly, NA has a similar effect on chromatin structure in GB cells.

### Dopamine

DA stimulates metabolic plasticity in GB, and it has been observed that the GB cells synthesize and secrete this catecholamine [17]. Using the intracranial injection of the HK-308-Luc or HK-374-Luc (which express luciferase) human GB cell lines for inducing tumor formation in immunosuppressed mice and followed by irradiation and subsequent subcutaneously (s.c.) administration of trifluoperazine [(TFP), an antagonist of D1 and D2 dopaminergic receptors], the tumor growth decreased and animal survival slightly improved. Interestingly, in the model mentioned above, radiation (8 Gy) stimulated the self-renewal of CSCs. Moreover, treatment with TFP reduces the expression of *Sox2, Oct4, Klf4, c-Myc*, and transcription factors involved in the stemness feature [64].

Glioma spheres derived from human GB tumors exposed to 7-OH-DPAT, an agonist of DA receptors, increased the number of cells. Interestingly, when the DA receptor type 2 (DRD2) was knocked down in GB cells, a decrease in the phosphorylation of STAT3, ERK, NANOG, and SOX2 expression was found [65], suggesting that one of the DA functions is to maintain a stem cell pool and therefore, participates in tumor recurrence.

In the patient-derived xenograft glioma line GBM43, the treatment with TMZ or radiation (2 Gy) increased the abundance of the H3K27ac mark in the promoter of DRD2 receptor gene. Conversely, orthotopic xenografts resulted in elevated expression of the human DRD2 receptor in the brains of nude (v/v) mice [17]. Following radiation treatment, the observed augmentation of H3K27ac presence in the DRD2 receptor promoter suggests a potential chromatin remodeling mechanism. It is well-established that transcriptionally active regions are more susceptible to impairment by ionizing radiation, and chromatin remodeling plays a crucial role in therapy [66].

The treatment of the human-derived GB cell line (T98 cell line) with an antagonist of the DA receptor D_4_R decreased cell viability compared to TMZ exposure [67]. Several studies suggest that DA acts as an autocrine regulator of GB development and a chromatin modifier by increasing the presence of H3K27ac, a permissive mark for gene expression (Fig. 2). The treatment with DA antagonists could become an alternative or coadjutant to conventional therapies for GB.

### Serotonin

In 2019, for the first time, it was observed that 5-HT can be covalently bound to histones, specifically the H3K4me3 [29]. Furthermore, this “serotonylation” mechanism in histones is related to permissive gene expression and neuronal differentiation [29]. While in GB, the GSCs exhibit the capacity for aberrant neuronal differentiation [68], a process that could be influenced by 5-HT acting as a PTM; however, causal studies to prove this hypothesis are needed.

The function of 5-HT in GB progression has been controversial. An orthotopic model inoculated with GBM39 human cell line neurospheres in mice, treated with TMZ and FLX increased apoptosis [20, 69]. The overexpression of tryptophan hydroxylase 1 (TPH1), an enzyme limiting peripheral 5-HT synthesis [70], in two cellular lines derived from human glioblastomas (LN229 and T98G), increased proliferation, migration, and expression of genes involved in cellular adhesion, while diminishing apoptosis. Thus, TPH1 expression stimulates proliferation and motility in glioma cells [71]. It is worth mentioning that LN229 and T98G possess different karyotypes, with LN229 being XX and T98G being XY, but the effects of TPH1 overexpression are conserved between the cellular lines (Fig. 2).

Metabolomic characterization of tissue samples from patients diagnosed with GB using Orbitrap secondary ion mass spectrometry (OrbiSIMS) technique shows that tumors are constituted by viable, necrotic, and non-cancerous areas where the distribution of metabolites across these regions is different. In turn, the 5-HT is present in all areas but is found in a more significant proportion in the necrotic section than in viable or non-cancerous regions [72]. Thus, 5-HT in GB could be involved in maintaining tumor cellular characteristics.

In GB human cell lines (LN229 and U251) treated with valeric acid, a partial agonist of the 5-HT_5A_ receptor, there is an increase in cleaved caspase 3 (CC3), an apoptosis marker, and LC3, which participates in the autophagy process [67]. Valeric acid also reduces cellular metabolism and colony formation in vitro, while in vivo, it reduces tumor growth when luciferase-LN229 and luciferase-U251 cells were subcutaneously implanted into nude mice [73].

Similarly, exposure of human GB-derived cells (U87 cell line) to combined treatment with imatinib, an anti-cancer drug that is a tyrosine kinase inhibitor, and FLX reduces the presence of a phosphorylated form of AKT (pAKT) and the percentage of proliferating cells [74]. Likewise, culturing human GB-derived cell lines (U87 or U251) with fluvoxamine, an SSRI, reduces pAKT and decreases migration and invasion by inhibiting actin polymerization. Organizing actin in the cytoskeleton is essential to cancer cell migration and invasion [75]. Thus, further research is required on the role of the serotoninergic system in GB.

In GB, the gene expression profile recapitulates the typical pattern of expression seen in human fetal neural progenitor cells (NPC) and their response under the stimulus of the 5-HT-1A receptor [76]. Activation of this receptor with its agonist, p-amino phenethyl-m-trifluoromethylphenyl piperazine (PAPP), inhibits NPC and GB expansion. Hence, it is hypothesized that GB recapitulates the typical pattern of NPC expression, supporting the parallelism between stemness and GB progression.

## Concluding and future directions

GSCs are recognized as the main impediment to an effective GB treatment due to their high capacity for self-renewal and therapy resistance. GSCs utilize various strategies to maintain their population within different niches of the GB, exhibiting proliferative and quiescent behavior. Alterations in the histone variant H2A.Z within GSC can profoundly impact their cellular programs, which are essential for maintaining their unique phenotype. These changes could be induced by neurotransmitters such as NA, DA, and 5-HT, which are widely distributed throughout the CNS under normal and pathological conditions, including GBs. Recent discoveries underscore the role of 5-HT and DA as chromatin modifiers and offer insight into potential involvement in regulating GSC behavior in the context of GB. However, rigorous studies are warranted to validate the influence of neurotransmitters, particularly regarding the H2A.Z histone.

## Data Availability

No datasets were generated or analysed during the current study.

## Abbreviations

5-HT: 5-Hydroxitriptamine
AKT2: AKT serine/threonine kinase 2
APOE: Apolipoprotein E
AQP4: Aquaporin-4
ASCL1: Achaete-scute family bHLH transcription factor 1
CASP1/4/5/8: Cysteine-aspartic acid protease 1/4/5/8
CC3: Cleaved caspase 3
CD15: Cluster of Differentiation 15 or stage-specific embryonic antigen-1 (SSEA-1)
CD24: Cluster of Differentiation 24
CD44: Cluster of Differentiation 44
CD9: Cluster of Differentiation 9
CHI3L: Chitinase-3-like protein 1
c-Myc: Cellular myelocytomatosis oncogene
CNS: Central nervous system
CRIPTO1: Teratocarcinoma-Derived Growth Factor-1 (Cripto-1)
DA: Dopamine
DCX: Doublecortin
DLL3: Delta-like ligand 3
DPPA2: Developmental pluripotency-associated 2
DRD2: Dopamine receptor type 2
EGFR: Epidermal growth factor receptor
EMT: Epithelial-mesenchymal-transition
ERBB3: Receptor tyrosine-protein kinase 3
ERK: Extracellular signal-regulated kinase
FGFR3: Fibroblast growth factor receptor 3
FLX: Fluoxetine
GB: Glioblastoma
GFAP: Glial fibrillary acidic protein
GSCs: glioma stem cells
H3K27ac: Histone H3 Lysine 27 acetylation
H3K4me1: Monomethylation of Histone H3 at Lysine 4
H3K4me3: Trimethylation of Histone H3 at Lysine 4
HIF: Hypoxia-inducible factor
ILR4: Interleukin 4 receptor
KLF4: Krüppel-like factor 4
LC3: Microtubule-associated protein 1 A/1B-light chain 3
MAPT: Microtubule-associated protein tau
MMP-11: Matrix metallopeptidase-11
NA: Noradrenaline
NANOG: NANOG homeobox
NES: Nestin
NKX2-2: NK2 Homeobox 2
NPC: Neural progenitor cells
OLIG2: Oligodendrocyte transcription factor 2
OLIG2: Oligodendrocyte transcription factor 2
P2RY1: Purinergic receptor P2Y1
pAKT: Phosphorylated form of AKT
PAPP: p-amino phenethyl-m-trifluoromethylphenyl piperazine
PDGFA: Platelet-derived growth factor subunit A
PROM1: Prominin-1 or CD133
PTMs: Post-translational modifications
PVN: Perivascular niche
RELB: RELB Proto-Oncogene, NF-KB Subunit
SOX11: SRY-box transcription factor 11
SOX2: SRY-box transcription factor 2
SOX4: SRY-box transcription factor 4
STAT3: Signal transducer and activator of transcription 3
TFP: Trifluoperazine
TGM2: Transglutaminase 2
TLR2: Toll-Like Receptor 2
TLR4: Toll-Like Receptor 4
TMZ: Temozolomide
TPH1: Tryptophan hydroxylase 1
TRADD: Tumor necrosis factor receptor type 1-associated DEATH domain protein
TSS: Transcription start site
VIM: Vimentin

## References

[1] Das S, Srikanth M, Kessler JA (2008) Nat Clin Pract Neurol 4:427–435. 10.1038/ncpneuro0862 18628751 10.1038/ncpneuro0862

[2] Geribaldi-Doldan N, Fernandez-Ponce C, Quiroz RN, Sanchez-Gomar I, Escorcia LG, Velasquez EP, Quiroz EN (2020) Front Oncol 10:603495. 10.3389/fonc.2020.603495 33585220 10.3389/fonc.2020.603495 PMC7879977

[3] Walker FM, Sobral LM, Danis E, Sanford B, Donthula S, Balakrishnan I, Wang D, Pierce A, Karam SD, Kargar S, Serkova NJ, Foreman NK, Venkataraman S, Dowell R, Vibhakar R, Dahl NA (2024) Nat Commun 15:4616. 10.1038/s41467-024-48214-3 38816355 10.1038/s41467-024-48214-3 PMC11139976

[4] Bao S, Wu Q, McLendon RE, Hao Y, Shi Q, Hjelmeland AB, Dewhirst MW, Bigner DD, Rich JN (2006) Nature 444:756–760. 10.1038/nature05236 17051156 10.1038/nature05236

[5] Huang M, Zhang D, Wu JY, Xing K, Yeo E, Li C, Zhang L, Holland E, Yao L, Qin L, Binder ZA, O’Rourke DM, Brem S, Koumenis C, Gong Y, Fan Y (2020) Sci Transl Med 12. 10.1126/scitranslmed.aay7522 10.1126/scitranslmed.aay7522 PMC7261487 32102932

[6] Mauvais-Jarvis F, Bairey Merz N, Barnes PJ, Brinton RD, Carrero JJ, DeMeo DL, De Vries GJ, Epperson CN, Govindan R, Klein SL, Lonardo A, Maki PM, McCullough LD, Regitz-Zagrosek V, Regensteiner JG, Rubin JB, Sandberg K, Suzuki A (2020) Lancet 396:565–582. 10.1016/S0140-6736(20)31561-0 32828189 10.1016/S0140-6736(20)31561-0 PMC7440877

[7] Yang W, Warrington NM, Taylor SJ, Whitmire P, Carrasco E, Singleton KW, Wu N, Lathia JD, Berens ME, Kim AH, Barnholtz-Sloan JS, Swanson KR, Luo J, Rubin JB (2019) Sci Transl Med 11. 10.1126/scitranslmed.aao5253 10.1126/scitranslmed.aao5253 PMC6502224 30602536

[8] Carrano A, Juarez JJ, Incontri D, Ibarra A (2021) H Guerrero Cazares Cells 10. 10.3390/cells10071783 10.3390/cells10071783 PMC8303471 34359952

[9] Pina-Medina AG, Diaz NF, Molina-Hernandez A, Mancilla-Herrera I, Camacho-Arroyo I (2020) Life Sci 249:117536. 10.1016/j.lfs.2020.117536 32165211 10.1016/j.lfs.2020.117536

[10] Lam KHB, Leon AJ, Hui W, Lee SC, Batruch I, Faust K, Klekner A, Hutoczki G, Koritzinsky M, Richer M, Djuric U, Diamandis P (2022) Nat Commun 13:116. 10.1038/s41467-021-27667-w 35013227 10.1038/s41467-021-27667-w PMC8748638

[11] Puchalski RB, Shah N, Miller J, Dalley R, Nomura SR, Yoon JG, Smith KA, Lankerovich M, Bertagnolli D, Bickley K, Boe AF, Brouner K, Butler S, Caldejon S, Chapin M, Datta S, Dee N, Desta T, Dolbeare T, Dotson N, Ebbert A, Feng D, Feng X, Fisher M, Gee G, Goldy J, Gourley L, Gregor BW, Gu G, Hejazinia N, Hohmann J, Hothi P, Howard R, Joines K, Kriedberg A, Kuan L, Lau C, Lee F, Lee H, Lemon T, Long F, Mastan N, Mott E, Murthy C, Ngo K, Olson E, Reding M, Riley Z, Rosen D, Sandman D, Shapovalova N, Slaughterbeck CR, Sodt A, Stockdale G, Szafer A, Wakeman W, Wohnoutka PE, White SJ, Marsh D, Rostomily RC, Ng L, Dang C, Jones A, Keogh B, Gittleman HR, Barnholtz-Sloan JS, Cimino PJ, Uppin MS, Keene CD, Farrokhi FR, Lathia JD, Berens ME, Iavarone A, Bernard A, Lein E, Phillips JW, Rostad SW (2018) C. Cobbs, M.J. Hawrylycz and G.D. Foltz, Science 360, 660–663 10.1126/science.aaf2666 10.1126/science.aaf2666 PMC6414061 29748285

[12] Verhaak RG, Hoadley KA, Purdom E, Wang V, Qi Y, Wilkerson MD, Miller CR, Ding L, Golub T, Mesirov JP, Alexe G, Lawrence M, O’Kelly M, Tamayo P, Weir BA, Gabriel S, Winckler W, Gupta S, Jakkula L, Feiler HS, Hodgson JG, James CD, Sarkaria JN, Brennan C, Kahn A, Spellman PT, Wilson RK, Speed TP, Gray JW, Meyerson M, Getz G, Perou CM (2010) Hayes and N. Cancer Genome Atlas Research. Cancer Cell 17:98–110. 10.1016/j.ccr.2009.12.020 20129251 10.1016/j.ccr.2009.12.020 PMC2818769

[13] Ikeda Y, Nakazawa S (1984) Nihon Ika Daigaku Zasshi 51:132–133. 10.1272/jnms1923.51.132 6725540 10.1272/jnms1923.51.132

[14] Anastasaki C, Gao Y, Gutmann DH (2023) Dev Cell 58:81–93. 10.1016/j.devcel.2022.12.011 36693322 10.1016/j.devcel.2022.12.011 PMC9883043

[15] Krishna S, Choudhury A, Keough MB, Seo K, Ni L, Kakaizada S, Lee A, Aabedi A, Popova G, Lipkin B, Cao C, Nava Gonzales C, Sudharshan R, Egladyous A, Almeida N, Zhang Y, Molinaro AM, Venkatesh HS, Daniel AGS, Shamardani K, Hyer J, Chang EF, Findlay A, Phillips JJ, Nagarajan S, Raleigh DR, Brang D, Monje M, Hervey-Jumper SL (2023) Nature 617:599–607. 10.1038/s41586-023-06036-1 37138086 10.1038/s41586-023-06036-1 PMC10191851

[16] Venkatesh HS, Morishita W, Geraghty AC, Silverbush D, Gillespie SM, Arzt M, Tam LT, Espenel C, Ponnuswami A, Ni L, Woo PJ, Taylor KR, Agarwal A, Regev A, Brang D, Vogel H, Hervey-Jumper S, Bergles DE, Suva ML, Malenka RC, Monje M (2019) Nature 573:539–545. 10.1038/s41586-019-1563-y 31534222 10.1038/s41586-019-1563-y PMC7038898

[17] Caragher SP, Shireman JM, Huang M, Miska J, Atashi F, Baisiwala S, Hong Park C, Saathoff MR, Warnke L, Xiao T, Lesniak MS, James CD, Meltzer H, Tryba AK, Ahmed AU (2019) J Neurosci 39:1982–1993. 10.1523/JNEUROSCI.1589-18.2018 30651332 10.1523/JNEUROSCI.1589-18.2018 PMC6507082

[18] Vo VTA, Kim S, Hua TNM, Oh J, Jeong Y (2022) Commun Biol 5:593. 10.1038/s42003-022-03538-y 35710828 10.1038/s42003-022-03538-y PMC9203457

[19] Yang K, Xu R, Le W (2021) Oncol Rep 45. 10.3892/or.2021.8025 10.3892/or.2021.8025 33760205

[20] Bi J, Khan A, Tang J, Armando AM, Wu S, Zhang W, Gimple RC, Reed A, Jing H, Koga T, Wong IT, Gu Y, Miki S, Yang H, Prager B, Curtis EJ, Wainwright DA, Furnari FB, Rich JN, Cloughesy TF, Kornblum HI, Quehenberger O, Rzhetsky A, Cravatt BF, Mischel PS (2021) Cell Rep 37:109957. 10.1016/j.celrep.2021.109957 34731610 10.1016/j.celrep.2021.109957 PMC8856626

[21] Bi J, Chowdhry S, Wu S, Zhang W, Masui K, Mischel PS (2020) Nat Rev Cancer 20:57–70. 10.1038/s41568-019-0226-5 31806884 10.1038/s41568-019-0226-5

[22] Bagert JD, Mitchener MM, Patriotis AL, Dul BE, Wojcik F, Nacev BA, Feng L, Allis CD, Muir TW (2021) Nat Chem Biol 17:403–411. 10.1038/s41589-021-00738-1 33649601 10.1038/s41589-021-00738-1 PMC8174649

[23] Chan JC, Maze I (2020) Trends Biochem Sci 45:829–844. 10.1016/j.tibs.2020.05.009 32498971 10.1016/j.tibs.2020.05.009 PMC7502514

[24] Millan-Zambrano G, Burton A, Bannister AJ, Schneider R (2022) Nat Rev Genet 23:563–580. 10.1038/s41576-022-00468-7 35338361 10.1038/s41576-022-00468-7

[25] Kurumizaka H, Kujirai T, Takizawa Y (2021) J Mol Biol 433:166678. 10.1016/j.jmb.2020.10.012 33065110 10.1016/j.jmb.2020.10.012

[26] Schwartzentruber J, Korshunov A, Liu XY, Jones DT, Pfaff E, Jacob K, Sturm D, Fontebasso AM, Quang DA, Tonjes M, Hovestadt V, Albrecht S, Kool M, Nantel A, Konermann C, Lindroth A, Jager N, Rausch T, Ryzhova M, Korbel JO, Hielscher T, Hauser P, Garami M, Klekner A, Bognar L, Ebinger M, Schuhmann MU, Scheurlen W, Pekrun A, Fruhwald MC, Roggendorf W, Kramm C, Durken M, Atkinson J, Lepage P, Montpetit A, Zakrzewska M, Zakrzewski K, Liberski PP, Dong Z, Siegel P, Kulozik AE, Zapatka M, Guha A, Malkin D, Felsberg J, Reifenberger G, von Deimling A, Ichimura K, Collins VP, Witt H, Milde T, Witt O, Zhang C, Castelo-Branco P, Lichter P, Faury D, Tabori U, Plass C, Majewski J (2012) S.M. Pfister and N. Jabado, Nature 482, 226–231 10.1038/nature10833

[27] Liu I, Jiang L, Samuelsson ER, Marco Salas S, Beck A, Hack OA, Jeong D, Shaw ML, Englinger B, LaBelle J, Mire HM, Madlener S, Mayr L, Quezada MA, Trissal M, Panditharatna E, Ernst KJ, Vogelzang J, Gatesman TA, Halbert ME, Palova H, Pokorna P, Sterba J, Slaby O, Geyeregger R, Diaz A, Findlay IJ, Dun MD, Resnick A, Suva ML, Jones DTW, Agnihotri S, Svedlund J, Koschmann C, Haberler C, Czech T, Slavc I, Cotter JA, Ligon KL, Alexandrescu S, Yung WKA, Arrillaga-Romany I, Gojo J, Monje M, Nilsson M, Filbin MG (2022) Nat Genet 54:1881–1894. 10.1038/s41588-022-01236-3 36471067 10.1038/s41588-022-01236-3 PMC9729116

[28] Wu Y, Fletcher M, Gu Z, Wang Q, Costa B, Bertoni A, Man KH, Schlotter M, Felsberg J, Mangei J, Barbus M, Gaupel AC, Wang W, Weiss T, Eils R, Weller M, Liu H, Reifenberger G, Korshunov A, Angel P, Lichter P, Herrmann C, Radlwimmer B (2020) Nat Commun 11:6434. 10.1038/s41467-020-20225-w 33339831 10.1038/s41467-020-20225-w PMC7749178

[29] Farrelly LA, Thompson RE, Zhao S, Lepack AE, Lyu Y, Bhanu NV, Zhang B, Loh YE, Ramakrishnan A, Vadodaria KC, Heard KJ, Erikson G, Nakadai T, Bastle RM, Lukasak BJ, Zebroski H 3rd, Alenina N, Bader M, Berton O, Roeder RG, Molina H, Gage FH, Shen L, Garcia BA, Li H, Muir TW, Maze I (2019) Nature 567:535–539. 10.1038/s41586-019-1024-7 10.1038/s41586-019-1024-7 PMC6557285 30867594

[30] Lepack AE, Werner CT, Stewart AF, Fulton SL, Zhong P, Farrelly LA, Smith ACW, Ramakrishnan A, Lyu Y, Bastle RM, Martin JA, Mitra S, O’Connor RM, Wang ZJ, Molina H, Turecki G, Shen L, Yan Z, Calipari ES, Dietz DM, Kenny PJ, Maze I (2020) Science 368:197–201. 10.1126/science.aaw8806 32273471 10.1126/science.aaw8806 PMC7228137

[31] Lukasak BJ, Mitchener MM, Kong L, Dul BE, Lazarus CD, Ramakrishnan A, Ni J, Shen L, Maze I, Muir TW (2022) Proc Natl Acad Sci U S A 119. 10.1073/pnas.2208672119. e2208672119 10.1073/pnas.2208672119 PMC9618071 36256821

[32] Hambardzumyan D, Bergers G (2015) Trends Cancer 1:252–265. 10.1016/j.trecan.2015.10.009 27088132 10.1016/j.trecan.2015.10.009 PMC4831073

[33] Aderetti DA, Hira VVV, Molenaar RJ, van Noorden CJF (2018) Biochim Biophys Acta Rev Cancer 1869:346–354. 10.1016/j.bbcan.2018.04.008 29684521 10.1016/j.bbcan.2018.04.008

[34] Soeda A, Park M, Lee D, Mintz A, Androutsellis-Theotokis A, McKay RD, Engh J, Iwama T, Kunisada T, Kassam AB, Pollack IF, Park DM (2009) Oncogene 28:3949–3959. 10.1038/onc.2009.252 19718046 10.1038/onc.2009.252

[35] Benedetti V, Banfi F, Zaghi M, Moll-Diaz R, Massimino L, Argelich L, Bellini E, Bido S, Muggeo S, Ordazzo G, Mastrototaro G, Moneta M, Sessa A, Broccoli V (2022) Sci Adv 8:eabn3986. 10.1126/sciadv.abn3986 35921410 10.1126/sciadv.abn3986 PMC9348799

[36] Ye C, Li H, Li Y, Zhang Y, Liu G, Mi H, Li H, Xiao Q, Niu L, Yu X (2022) iScience 25:104872. 10.1016/j.isci.2022.104872 36034219 10.1016/j.isci.2022.104872 PMC9399482

[37] Ricci-Vitiani L, Pallini R, Biffoni M, Todaro M, Invernici G, Cenci T, Maira G, Parati EA, Stassi G, Larocca LM (2010) R De Maria Nat 468:824–828. 10.1038/nature09557 10.1038/nature09557 21102434

[38] Wang R, Chadalavada K, Wilshire J, Kowalik U, Hovinga KE, Geber A, Fligelman B, Leversha M, Brennan C, Tabar V (2010) Nature 468:829–833. 10.1038/nature09624 21102433 10.1038/nature09624

[39] Couturier CP, Ayyadhury S, Le PU, Nadaf J, Monlong J, Riva G, Allache R, Baig S, Yan X, Bourgey M, Lee C, Wang YCD, Wee Yong V, Guiot MC, Najafabadi H, Misic B, Antel J, Bourque G, Ragoussis J, Petrecca K (2020) Nat Commun 11:3406. 10.1038/s41467-020-17186-5 32641768 10.1038/s41467-020-17186-5 PMC7343844

[40] Liau B.B., Sievers C., Donohue L.K., Gillespie S.M., Flavahan W.A., Miller T.E., Venteicher A.S., Hebert C.H., Carey C.D., Rodig S.J., Shareef S.J., Najm F.J., van Galen P., Wakimoto H., Cahill D.P., Rich J.N., Aster J.C., Suva M.L., Patel A.P., Bernstein B.E. (2017) Cell Stem Cell 20:233–246. 10.1016/j.stem.2016.11.003. e237 10.1016/j.stem.2016.11.003 PMC5291795 27989769

[41] Rehfeld M, Matschke J, Hagel C, Willenborg K, Glatzel M, Bernreuther C (2021) J Cancer Res Clin Oncol 147:2969–2982. 10.1007/s00432-021-03704-5 34170383 10.1007/s00432-021-03704-5 PMC8397690

[42] Antonica F, Santomaso L, Pernici D, Petrucci L, Aiello G, Cutarelli A, Conti L, Romanel A, Miele E, Tebaldi T, Tiberi L (2022) Nat Commun 13:4767. 10.1038/s41467-022-32448-0 35970913 10.1038/s41467-022-32448-0 PMC9378633

[43] Xie XP, Laks DR, Sun D, Ganbold M, Wang Z, Pedraza AM, Bale T, Tabar V, Brennan C, Zhou X, Parada LF (2022) Dev Cell 57:32–46. 10.1016/j.devcel.2021.12.007. e38 35016005 10.1016/j.devcel.2021.12.007 PMC8820651

[44] Wang P, Zhao L, Gong S, Xiong S, Wang J, Zou D, Pan J, Deng Y, Yan Q, Wu N, Liao B (2021) Cell Death Dis 12:312. 10.1038/s41419-021-03598-8 33762574 10.1038/s41419-021-03598-8 PMC7990922

[45] Vinel C, Rosser G, Guglielmi L, Constantinou M, Pomella N, Zhang X, Boot JR, Jones TA, Millner TO, Dumas AA, Rakyan V, Rees J, Thompson JL, Vuononvirta J, Nadkarni S, El Assan T, Aley N, Lin YY, Liu P, Nelander S, Sheer D, Merry CLR, Marelli-Berg F, Brandner S, Marino S (2021) Nat Commun 12:6130. 10.1038/s41467-021-26297-6 34675201 10.1038/s41467-021-26297-6 PMC8531305

[46] Lu X, Maturi NP, Jarvius M, Yildirim I, Dang Y, Zhao L, Xie Y, Tan EJ, Xing P, Larsson R, Fryknas M, Uhrbom L, Chen X (2022) Nat Commun 13:2236. 10.1038/s41467-022-29912-2 35469026 10.1038/s41467-022-29912-2 PMC9038925

[47] Myers B.L., Brayer K.J., Paez-Beltran L.E., Keith M.S., Suzuki H., Newville J., Anderson R.H., Lo Y., Mertz C.M., Kollipara R., Borromeo M.D., Bachoo R.M., Johnson J.E., Vue T.Y. (2023) bioRxiv. 10.1101/2023.09.30.560206

[48] Zhao K, Cui X, Wang Q, Fang C, Tan Y, Wang Y, Yi K, Yang C, You H, Shang R, Wang J, Kang C (2019) Cell Death Dis 10:877. 10.1038/s41419-019-2108-x 31754093 10.1038/s41419-019-2108-x PMC6872557

[49] Stepniak K, Machnicka MA, Mieczkowski J, Macioszek A, Wojtas B, Gielniewski B, Poleszak K, Perycz M, Krol SK, Guzik R, Dabrowski MJ, Draminski M, Jardanowska M, Grabowicz I, Dziedzic A, Kranas H, Sienkiewicz K, Diamanti K, Kotulska K, Grajkowska W, Roszkowski M, Czernicki T, Marchel A, Komorowski J, Kaminska B, Wilczynski B (2021) Nat Commun 12:3621. 10.1038/s41467-021-23922-2 34131149 10.1038/s41467-021-23922-2 PMC8206121

[50] Yoon J, Grinchuk OV, Tirado-Magallanes R, Ngian ZK, Tay EXY, Chuah YH, Lee BWL, Feng J, Crasta KC, Ong CT, Benoukraf T, Ong DST (2022) Cell Death Differ 29:1379–1394. 10.1038/s41418-021-00926-5 35058574 10.1038/s41418-021-00926-5 PMC9287453

[51] Colino-Sanguino Y, Clark SJ, Valdes-Mora F (2022) Trends Genet 38:273–289. 10.1016/j.tig.2021.10.003 34702577 10.1016/j.tig.2021.10.003

[52] Domaschenz R, Kurscheid S, Nekrasov M, Han S, Tremethick DJ (2017) Cell Rep 21:943–952. 10.1016/j.celrep.2017.09.086 29069602 10.1016/j.celrep.2017.09.086

[53] Roche J (2018) Cancers (Basel) 10. 10.3390/cancers10020052

[54] Avila-Lopez PA, Guerrero G, Nunez-Martinez HN, Peralta-Alvarez CA, Hernandez-Montes G, Alvarez-Hilario LG, Herrera-Goepfert R, Albores-Saavedra J, Villegas-Sepulveda N, Cedillo-Barron L, Montes-Gomez AE, Vargas M, Schnoor M, Recillas-Targa F, Hernandez-Rivas R (2021) Oncogene 40:2065–2080. 10.1038/s41388-021-01664-1 33627784 10.1038/s41388-021-01664-1 PMC7979544

[55] Yang B, Tong R, Liu H, Wu J, Chen D, Xue Z, Ding C, Zhou L, Xie H, Wu J, Zheng S (2018) Int J Oncol 52:1235–1245. 10.3892/ijo.2018.4292 29532867 10.3892/ijo.2018.4292 PMC5843396

[56] Avila-Lopez PA, Nunez-Martinez HN, Peralta-Alvarez CA, Martinez-Calvillo S, Recillas-Targa F, Hernandez-Rivas R (2022) Arch Med Res 53:840–858. 10.1016/j.arcmed.2022.11.010 36470770 10.1016/j.arcmed.2022.11.010

[57] Kim K, Punj V, Choi J, Heo K, Kim JM, Laird PW, An W (2013) Epigenetics Chromatin 6:34. 10.1186/1756-8935-6-34 24279307 10.1186/1756-8935-6-34 PMC3853418

[58] Newburgh RW, Rosenberg RN (1972) Proc Natl Acad Sci U S A 69:1677–1680. 10.1073/pnas.69.7.1677 4340154 10.1073/pnas.69.7.1677 PMC426776

[59] Stojic J, Hagemann C, Haas S, Herbold C, Kuhnel S, Gerngras S, Roggendorf W, Roosen K, Vince GH (2008) Neurosci Res 60:40–49. 10.1016/j.neures.2007.09.009 17980449 10.1016/j.neures.2007.09.009

[60] Zhong J, Shan W, Zuo Z (2021) Brain Res 1756:147280. 10.1016/j.brainres.2021.147280 33515535 10.1016/j.brainres.2021.147280 PMC7904089

[61] Izadpanah MH, Forghanifard MM (2022) Genes (Basel) 13. 10.3390/genes13122369 10.3390/genes13122369 PMC9777594 36553636

[62] Wang X, Wang Y, Xie F, Song ZT, Zhang ZQ, Zhao Y, Wang SD, Hu H, Zhang YS, Qian LJ (2022) BMC Cancer 22:213. 10.1186/s12885-022-09330-9 35219305 10.1186/s12885-022-09330-9 PMC8882280

[63] Maity S, Jarome TJ, Blair J, Lubin FD, Nguyen PV (2016) J Physiol 594:863–881. 10.1113/JP271432 26574176 10.1113/JP271432 PMC4753274

[64] Bhat K, Saki M, Vlashi E, Cheng F, Duhachek-Muggy S, Alli C, Yu G, Medina P, He L, Damoiseaux R, Pellegrini M, Zemke NR, Nghiemphu PL, Cloughesy TF, Liau LM, Kornblum HI, Pajonk F (2020) Proc Natl Acad Sci U S A 117:11085–11096. 10.1073/pnas.1920154117 32358191 10.1073/pnas.1920154117 PMC7245100

[65] Jeon HM, Oh YT, Shin YJ, Chang N, Kim D, Woo D, Yeup Y, Joo KM, Jo H, Yang H, Lee JK, Kang W, Sa J, Lee WJ, Hale J, Lathia JD, Purow B, Park MJ, Park JB, Nam DH, Lee J (2023) Neoplasia 39:100894. 10.1016/j.neo.2023.100894 36972629 10.1016/j.neo.2023.100894 PMC10066565

[66] Brambilla F, Garcia-Manteiga JM, Monteleone E, Hoelzen L, Zocchi A, Agresti A, Bianchi ME (2020) Nucleic Acids Res 48:8993–9006. 10.1093/nar/gkaa613 32710624 10.1093/nar/gkaa613 PMC7498322

[67] Pavletic P, Semeano A, Yano H, Bonifazi A, Giorgioni G, Piergentili A, Quaglia W, Sabbieti MG, Agas D, Santoni G, Pallini R, Ricci-Vitiani L, Sabato E, Vistoli G, Del Bello F (2022) J Med Chem 65:12124–12139. 10.1021/acs.jmedchem.2c00840 36098685 10.1021/acs.jmedchem.2c00840 PMC9511495

[68] Beier CP, Rasmussen T, Dahlrot RH, Tenstad HB, Aaro JS, Sorensen MF, Heimisdottir SB, Sorensen MD, Svenningsen P, Riemenschneider MJ, Beier D, Kristensen BW (2018) Sci Rep 8:14965. 10.1038/s41598-018-33282-5 30297697 10.1038/s41598-018-33282-5 PMC6175915

[69] Rogakou EP, Nieves-Neira W, Boon C, Pommier Y, Bonner WM (2000) J Biol Chem 275:9390–9395. 10.1074/jbc.275.13.9390 10734083 10.1074/jbc.275.13.9390

[70] McKinney J, Knappskog PM, Haavik J (2005) J Neurochem 92:311–320. 10.1111/j.1471-4159.2004.02850.x 15663479 10.1111/j.1471-4159.2004.02850.x

[71] Zhang J, Guo Z, Xie Q, Zhong C, Gao X, Yang Q (2022) BMC Cancer 22:457. 10.1186/s12885-022-09569-2 35473609 10.1186/s12885-022-09569-2 PMC9044587

[72] He W, Edney MK, Paine SML, Griffiths RL, Scurr DJ, Rahman R, Kim DH (2023) Anal Chem 95:5994–6001. 10.1021/acs.analchem.2c05807 36995369 10.1021/acs.analchem.2c05807 PMC10100400

[73] Lu Q, Ding Y, Li Y, Lu Q (2020) Int J Biol Sci 16:2104–2115. 10.7150/ijbs.44906 32549758 10.7150/ijbs.44906 PMC7294948

[74] Tzadok S, Beery E, Israeli M, Uziel O, Lahav M, Fenig E, Gil-Ad I, Weizman A, Nordenberg J (2010) Int J Oncol 37:1043–1051. 10.3892/ijo_00000756 20811727 10.3892/ijo_00000756

[75] Hayashi K, Michiue H, Yamada H, Takata K, Nakayama H, Wei FY, Fujimura A, Tazawa H, Asai A, Ogo N, Miyachi H, Nishiki T, Tomizawa K, Takei K, Matsui H (2016) Sci Rep 6:23372. 10.1038/srep23372 26988603 10.1038/srep23372 PMC4796892

[76] Diamandis P, Wildenhain J, Clarke ID, Sacher AG, Graham J, Bellows DS, Ling EK, Ward RJ, Jamieson LG, Tyers M, Dirks PB (2007). Chemical genetics reveals a complex functional ground state of neural stem cells. Nat Chem Biol 3(5) 268–273. 10.1038/nchembio873 10.1038/nchembio873 17417631

